# A multidisciplinary intervention to support return to work of individuals with breast cancer: a prospective feasibility study

**DOI:** 10.1007/s00520-026-11198-5

**Published:** 2026-09-16

**Authors:** Sara Paltrinieri, Luca Braglia, Francesca Bravi, Stefania Fugazzaro, Stefania Costi

**Affiliations:** 1 Research and EBP Unit, Health Professions Department, Azienda USL-IRCCS di Reggio Emilia, Via Giovanni Amendola N. 2, 42122 Reggio Emilia, Italy; 2 Public Health Sciences PhD Program, Department of Clinical Sciences and Community Health, Dipartimento di Eccellenza 2023-2027, University of Milan, Via Della Commenda N. 21, 20122 Milan, Italy; 3 Clinical Trials Center, Infrastructure Research and Statistics, Azienda USL-IRCCS di Reggio Emilia, Via Giovanni Amendola N. 2, 42122 Reggio Emilia, Italy; 4 Department of Clinical Sciences and Community Health, Dipartimento di Eccellenza 2023-2027, University of Milan, Via Della Commenda N. 21, 20122 Milan, Italy; 5 Physical Medicine and Rehabilitation Unit, Azienda USL-IRCCS di Reggio Emilia, Via Giovanni Amendola N. 2, 42122 Reggio Emilia, Italy; 6 Department of Surgery, Medicine, Dentistry and Morphological Sciences, University of Modena and Reggio Emilia, Via del Pozzo N. 71, 41124 Modena, Italy

**Keywords:** Breast neoplasms, Cancer survivors, Return to work, Vocational rehabilitation, Feasibility study

## Abstract

**Purpose:**

Work participation is one of the aims of cancer rehabilitation for individuals with breast cancer (BC). This study aimed to verify the feasibility of a multidisciplinary intervention addressing the work-related difficulties of working-age individuals with BC.

**Methods:**

In this prospective feasibility study, employed individuals with BC who reported work-related difficulties were eligible. Participants were assessed at baseline (T0), 1 month (T1), 3 months (T2), 6 months (T3), and 12 months (T4) after baseline. The feasibility of the intervention was determined by calculating the percentages of eligibility, recruitment, dropout, adherence to and satisfaction with the support received, and return to work.

**Results:**

Among 85 individuals, 32 (37.6%; 95% CI 27.4%–48.8%) met the eligibility criteria and were invited to participate, of whom 50% (95% CI 31.9%–68.1%) accepted. The highest dropout percentage was at T1 (12.5%; 95% CI 1.6%–38.3%). Adherence to the intervention was high throughout the study, with the highest percentage recorded at T1 (92.3%; 95% CI 64.0%–99.8%). At T4, all participants were satisfied with the intervention received. At T4, 10 participants out of 11 (95% CI 58.7%–99.8%) had achieved work reintegration. Occupational therapy was the most frequent intervention offered.

**Conclusion:**

This study offers valuable insights into the feasibility of complex interventions. The multidisciplinary intervention was tailored to individual needs, and it seemed feasible as it was proposed. By supporting individuals with BC facing work-related difficulties, these findings may inform the design of appropriate interventions, especially in terms of recruitment strategies, intervention characteristics, and appointment scheduling.

**Trial registration:**

ClinicalTrail.gov (ID NCT05309265, Registration date 25/03/2022)

**Supplementary Information:**

The online version contains supplementary material available at https://doi.org/10.1007/s00520-026-11198-5.

## Introduction

Work participation is one of the aims of cancer rehabilitation for individuals with breast cancer (BC).

In Italy, 436,000 new cancer diagnoses and over 1 million 5-year prevalent cases were recorded in 2022 [1]. BC diagnosis accounted for 13.2% of the incident cases, with 47.2% occurring in working-age individuals.

Cancer survivors are 1.4 times more likely to be unemployed than healthy individuals [2]. Among long-term survivors, 73% of those employed at diagnosis returned to work, but the percentage drops to 65% six years after diagnosis [3]. BC survivors are at increased risk of unemployment [2], with a return to work rate of 56% to 88% among those employed at diagnosis [4]. Despite improving survival [5], multimodal and prolonged treatments can cause adverse effects influencing work participation. Upper limb disability, including pain and lymphedema, may limit physically demanding tasks [6] and require accommodations [7]. Cancer-related fatigue and cognitive problems often contribute to prolonged absences or work discontinuation [8]. Accommodations to working conditions, including reduced work schedule or technological supports, may be beneficial [9], although not always feasible or sustainable increasing the risk of unemployment. As a matter of fact, strenuous posture and shift work were associated with a higher risk of not returning to work 2 years after BC diagnosis [10]. Limited work participation may also result from unsupportive workplaces [11] or excessive employer control [12].

Thus, individuals with BC at risk of work-related difficulties or unemployment should be identified early to provide tailored support. Individuals with BC may need information on employment rights and practical support [13, 14]. However, patients may hesitate to discuss these concerns with oncologists, and healthcare professionals typically address the topic only when raised by patients [15]. When work performance is affected by health or work conditions, accommodations were reported as facilitator of return to work [16]. Nevertheless, the work-related support for cancer survivors was not always satisfying [17, 18], negatively affecting work participation.

Recent literature indicates that multidisciplinary interventions consisting of vocational counseling, patient education, and physical exercises enhanced the return to work of cancer survivors as compared to standard care, with moderate quality of evidence [19]. One of the articles included in the systematic review of Algeo et al. [20] reported higher rates of return to work among BC survivors receiving a standardized multicomponent intervention consisting of physiotherapy, nutritional counseling, and spa treatment [21]. Nevertheless, to be effective, these approaches should be adapted to the context of application and to individual needs that could vary considerably [22].

Given the lack of data from Central and Southern Europe [23], we conducted a cross-sectional study to investigate the rate of return to work of cancer survivors in an Italian population [24]. Most participants returned to work, though half perceived difficulties related to mental and physical fatigue, task performance, and psychological distress [24, 25]. Based on these findings, in 2018, the Azienda USL–IRCCS of Reggio Emilia (Italy) developed the UNAMANO project, a social-healthcare pathway targeted at cancer survivors living in the Province of Reggio Emilia, which provides multidisciplinary support free of cost for patients for returning to work by addressing work-related difficulties [26]. By contacting the *In-Forma Salute* Service of the Azienda USL–IRCCS of Reggio Emilia, cancer survivors can receive one or more of the following types of support: information, occupational therapy, and social support.

Given the complex nature of this multidisciplinary intervention, a few years after its development, we decided to undertake a feasibility study to collect data that will help us refine patient selection procedures and develop a more appropriate intervention, making it easier to implement it in real-world settings.

Thus, the aim of this study was to verify the feasibility of the multidisciplinary intervention provided by the social-healthcare pathway of the UNAMANO project, aimed at addressing work-related difficulties in working-age individuals with BC.

## Methods

### Study design and setting

This prospective feasibility study was conducted at the Azienda USL–IRCCS of Reggio Emilia, Italy. As per standard care in the setting of implementation, individuals with BC who undergo sentinel or axillary lymph node removal are invited to participate in an educational outpatient group session, which is held approximately 2 weeks after surgery. This outpatient group session focuses on exercises to be performed progressively, and on precautions to be maintained regarding the operated breast and the ipsilateral upper limb, to prevent post-operative complications.

### Study population

Eligible patients were working-age individuals with BC (18–64 years) who had either returned to work or were planning to return and perceived work-related difficulties.

They were identified through a concurrent prospective observational single-cohort study [27] conceived and conducted by the same research team or among those referred to the UNAMANO project during the post-surgery educational session.

### Recruitment procedures

Between May 2022 and May 2023, working-age BC patients who attended the educational session were informed about and invited to participate in an observational study investigating employment status, work difficulties, and sick leave in employed individuals with BC and cancer survivorship needs perceived by individuals with BC regardless of their employment status [27]. For this observational study, potential participants were approached after the educational outpatient group session, where the study was presented and expressions of interest were collected. Eligible participants were adults diagnosed with BC who had attended the assigned session. Those interested were contacted by telephone approximately 3 days later to provide further information, answer questions, and schedule the baseline appointment. Participants in this observational study were prospectively followed-up until May 2024 to collect data regarding employment status, work difficulties, sick leave, and cancer survivorship needs. At each follow-up timepoint, the occupational therapist asked participants whether they perceived any work difficulties. If work-related issues emerged, participants were informed about the UNAMANO project [24] and invited to join also the related feasibility study. Potential participants for the feasibility study could be enrolled at any follow-up timepoint of the observational study.

Furthermore, participation in the feasibility study was also offered to eligible individuals who attended the outpatient group session but declined participation in the observational study. These patients were referred to the UNAMANO project and received a leaflet distributed during the educational session [25]. This method was adopted to ensure equitable access to the support provided by the UNAMANO project for all individuals with BC experiencing work-related difficulties, irrespective of their decision to participate in the observational study.

All participants in the feasibility study provided signed informed consent before baseline data collection.

### Data collection and assessment appointments

The recruitment period of this feasibility study lasted 24 months, between May 2022 and May 2024; the follow-up lasted 12 months until May 2025. During follow-up, participants were assessed five times: T0 (baseline), T1 (1 month after T0), T2 (3 months after T0), T3 (6 months after T0), and T4 (12 months after T0). Participants were given the option of attending follow-up appointments either in person or remotely by telephone.

### Outcomes

The feasibility of the multidisciplinary intervention was determined by calculating the percentage of eligibility, recruitment, dropout, adherence, satisfaction of the support received, and return to work/continuation of work, as described below:

- Eligibility: percentage of eligible individuals with BC who were invited to participate in the feasibility study among those recruited in the observational study
- Recruitment: percentage of individuals with BC who were included in the feasibility study among those eligible
- Dropout: percentage of participants who were lost at follow-up among those who attended the assessment appointments
- Adherence:
- Percentage of participants who complied with the multidisciplinary intervention among those who attended the assessment appointments
- Percentage of participants who complied with the intervention in the workplace among those to whom the occupational therapist proposed it
- Satisfaction: percentage of participants who perceived the intervention as supportive among those who attended the assessment appointment
- Return to work/continuation of work: percentage of participants who returned to work or continued to work among those who attended the assessment appointments

Table 1 shows when the outcomes were collected.

**Table 1:** Outcomes collected at each assessment appointment

	Timing				
	T0)	T1) T0 + 1 m	T2) T0 + 3 m	T3) T0 + 6 m	T4) T0 + 12 m
Outcomes					
a) Eligibility	X	-	-	-	-
b) Recruitment	X	-	-	-	-
c) Dropout	X	X	X	X	X
d) Adherence	-	X	X	X	X
e) Satisfaction	-	-	-	-	X
f) Return to work	X	X	X	X	X

### Information collected

Sociodemographic, work-related, and disease-related information were collected at baseline through a paper survey designed by the research team. Additionally, after the collection of this information, baseline included goal setting, multidisciplinary intervention proposal, and provision of the assessment appointment schedule by the occupational therapist. Work-related information (employment status and work-related difficulties) and disease-related information (type of treatment and rehabilitation) were collected at each of the subsequent assessment appointments. Work-related difficulties were categorized through the Core Set-VR-Onco, a checklist based on the International Classification of Functioning, Disability and Health [28] which was recently adapted and validated for a population of cancer survivors of the Province of Reggio Emilia [29, 30]. The information collected is described in the Supplementary material A (p. 1).

### Multidisciplinary intervention

The multidisciplinary intervention was tailored according to individual needs and may include one of the following modules of the UNAMANO project, or a combination of them, as reported in Table 2. A more detailed description of the multidisciplinary intervention is provided in the Supplementary material B (p. 2). Furthermore, the UNAMANO project integrates with the supportive care interventions (e.g., psycho-oncology, physical therapy, nutritional support) provided by the Azienda USL–IRCCS di Reggio Emilia. The various healthcare professionals following patients undergoing more than one supportive care intervention can discuss each case to gather more information and to coordinate support. Furthermore, when a specific need is detected, healthcare professionals can refer the patient to a specific supportive care intervention.

**Table 2:** Description of the multidisciplinary intervention

Modules	Aims	Professionals involved in the provision of the intervention	Intervention content	Assessment appointments
*Information*	To provide tailored information concerning work legal rights and to address participants’ work-related difficulties	- The information specialist-nurse responsible for the In-Forma Salute Service of the Azienda USL–IRCCS of Reggio Emilia - Labor union	- Assessment of participants’ work difficulties at baseline - Immediate provision of tailored information on identified needs - For work sector specific issues: targeted information search by the social healthcare pathway and involvement of other professionals of the social-healthcare pathway when needed	- Monitoring of employment status - Assessments of adherence to agreed intervention - Verify the occurrence of any further work-related difficulties and need for additional modules
*Occupational therapy*	To facilitate the return to work process through the provision of a rehabilitation intervention to overcome work-related difficulties in agreement with the employee, the employer, and the occupational physician	- Occupational therapist - Labor union	- Work activities description and identification of work-related difficulties at baseline - Provision of work accommodations - Workplace supervision, if accepted by the worker - Involvement of other professionals of the social-healthcare pathway when needed	- Monitoring of employment status - Assessments of adherence to agreed intervention - Verify the need to change the strategies of the intervention - Verify the occurrence of new work-related difficulties and need for additional modules
*Social support*	To find new job opportunities for those who have lost their jobs due to cancer	- Social cooperative	- Participant’s data were collected at baseline and shared with a social cooperative of the province - Provision of assistance in job search, prepare a curriculum vitae, conduct skill analysis, and professional training	- Monitoring of employment status - Assessments of compliance with agreements made with the cooperative

### Sample size

In absence of a priori hypotheses useful for a formal sample size justification, considering the early exploratory phase of the study, and being an embedded study of a previous observational study (for which 110–120 patients were planned to be recruited), for this study, a sample size of approximately 20 to 30 individuals with BC was adopted. The feasibility reasons behind this sample size were that we estimated that half of participants of the previous observational study would be of working age and employed at the time of diagnosis and, based on previous data [24], we estimated that half would have work difficulties.

### Statistical analysis

Among participants in the observational prospective study, the percentage of individuals who were invited to participate in the feasibility study (outcome eligibility) and those who accepted (outcome recruitment) were calculated for each follow-up timepoint. For each assessment appointment, the percentages of individuals with BC who dropped out from the study (outcome dropout), those who adhered to the proposed intervention, and accepted the intervention in the workplace (outcome adherence) were computed. To determine adherence, at the beginning of each appointment, participants were asked whether they had complied with what had been agreed on with the occupational therapist during the previous appointment, e.g., whether a participant had applied for disability, and/or had carried out the strategies agreed upon with the occupational therapist to overcome work difficulties. Adherence was assessed by the occupational therapist using a dichotomous response (“yes, adhered/no, did not adhere”). At T4, the percentage of participants who declared being satisfied with the intervention was calculated (outcome satisfaction). Satisfaction was assessed by using a dichotomous response (“yes, I am satisfied with the intervention/no, I am not satisfied with the intervention”). For each assessment appointment, the percentage of participants who had returned to work or continued to work was computed (outcome return to work/continuation of work). Return to work was defined as the occurrence of work reintegration, regardless of whether participants returned to their previous workplace or were employed by a different company, and irrespective of any workplace accommodations. Distributions of sociodemographic, work-related, and disease-related characteristics were provided for all individuals with BC who attended the baseline appointment.

Finally, 95% confidence intervals (CIs) were calculated for each of the percentages using the Clopper-Pearson method [31].

## Results

Of the cohort of 85 working-age individuals with BC recruited in the observational study, 32 (37.6% CI 27.4%–48.8%) were invited to participate in the feasibility study; of these, 16 (50.0% CI 31.9%–68.1%) accepted the invitation and signed the informed consent. Apart from the patients recruited through the observational study, no eligible patient contacted UNAMANO after being referred during the outpatient group session.

**Fig. 1:**
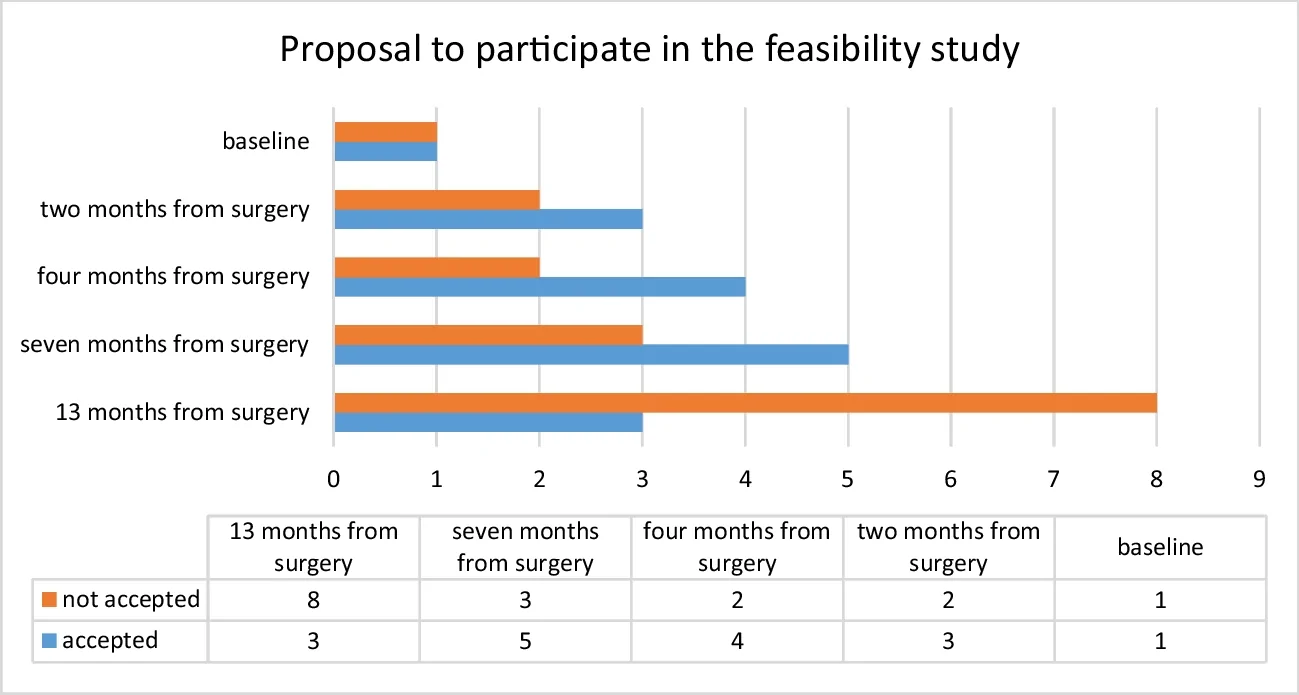
Invitation to participate in the feasibility study by each follow-up timepoint of the observational study

Figure 1 shows the number of participants to the observational study who were invited to participate also in the feasibility study and the number of those who were recruited at each follow-up timepoint of the observational study. As for eligibility, the highest number of invitations to participate in the feasibility study (n. 11) was made 13 months after surgery. As for recruitment, the highest percentage of participants was included in the feasibility study 4 months after surgery (66.7% CI 22.3%–95.7%). Table 3 reports the recruitment percentage by each follow-up timepoint of the observational study.

**Table 3:** Percentage of recruitment by each follow-up timepoint of the observational study

	***n***. of proposal	***n***. of recruit	% (95% CI)
Follow-up timepoint			
Total	32	16	50.0 (31.9–68.1)
Baseline	2	1	50.0 (1.3–98.7)
2 months from surgery	5	3	60.0 (14.7–94.7)
4 months from surgery	6	4	66.7 (22.3–95.7)
7 months from surgery	8	5	62.5 (24.5–91.5)
13 months from surgery	11	3	27.3 (6.0–61.0)

Supplementary material C (p. 3) reports the flow diagram of participants in the feasibility study. Overall, the number of participants who took part in the assessments was as follows: 14 at T0, 13 at T1, 12 at T2, 12 at T3, and 11 at T4.

Supplementary material D (p. 4) reports the information collected at baseline. All participants were female, and the mean age was 50.7 years (± 7.0). Most of the workers were employees (78.6%) in the private sector (85.7%) with permanent employment contract (71.4%). Having a psychologically or physically demanding job was reported by 64.3% and 50.0% of the participants, respectively.

Supplementary material E (p. 6) represents the treatment pathway from baseline to each assessment appointment.

**Fig. 2:**
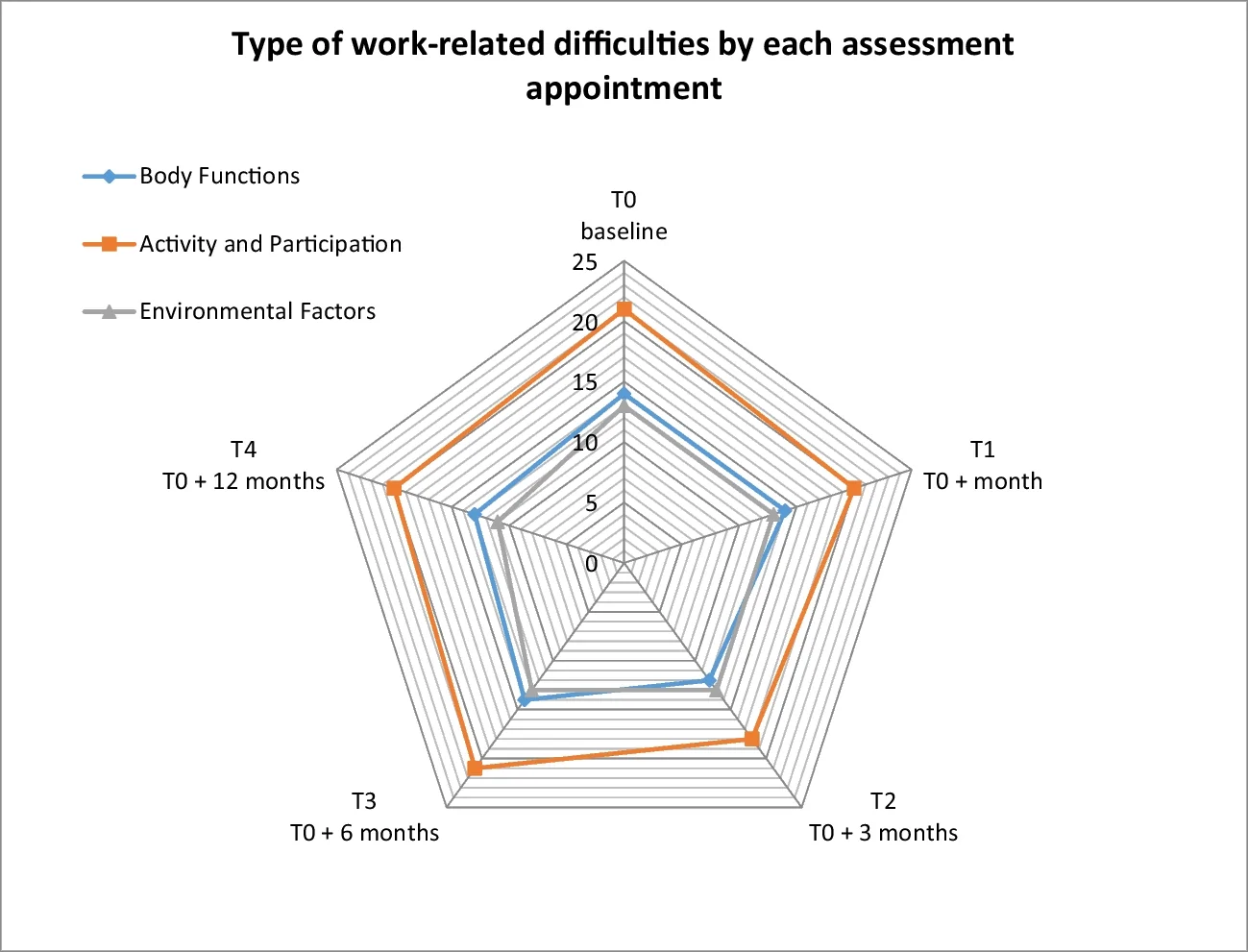
Absolute number and type of work difficulties reported at each assessment appointment

Figure 2 reports the absolute numbers of work difficulties, which were described through the ICF components of the CS-VR-Onco [29, 30]. Overall, T4 was the assessment appointment when fewer difficulties were reported. Supplementary F (p. 7) reports the absolute numbers of work difficulties described through the ICF.

Table 4 describes the study participants’ percentages of dropout, adherence, satisfaction, and return to work. The dropout percentage was 25% for the study. At T0, the highest percentage of dropout of participants (12.5% CI 1.6%–38.3%) was registered. One participant dropped out because of high emotional distress, the other because she did not want to commit to another year of appointments, after having attended all the follow-up timepoints of the observational study. Further dropouts were at T1 (n.1) and at T3 (n.1): the participant dropped out at T1 showed little confidence in the occupational therapy intervention that was proposed, while participant who dropped out at T3 found a new job after receiving support from her labor union and did not have time to come to the last assessment appointment. Very few missing data occurred: one participant missed the T2 and T4 assessment appointments. On average, the longest appointment was at T0 (52.9 min), which was held in person for all the participants; instead, the participants’ request to conduct assessment appointments remotely increased steadily over the course of the study (9 participants at T4).

**Table 4:** Percentage of outcomes to determine the feasibility of the multidisciplinary intervention by each assessment appointment

		Timepoint				
		T0)	T1) T0 + 1 m	T2) T0 + 3 m	T3) T0 + 6 m	T4) T0 + 12 m
Total participants		14	13	12	12	11
Outcomes						
c) Dropout	% (95% CI) of dropouts*	12.5 (1.6–38.3)	7.1 (0.2–33.9)	0.0 (0.0–26.5)	7.7 (0.2–36.0)	0.0 (0.0–28.5)
	*average meeting duration min. (SD)*	52.9 (30.4)	27.7 (12.4)	34.6 (14.1)	33.7 (11.5)	32.3 (12.1)
	*n. of meetings in person*	14	6	6	5	2
d) Adherence	*% (95% CI) of participants who complied with the intervention*	NA	92.3 (64.0–99.8)	91.7 (61.5–99.8)	75.0 (42.8–94.5)	90.9 (58.7–99.8)
	*% (95% CI) proposal of conducting the intervention in the workplace*	28.6 (8.4–58.1)	23.1 (5.0–53.8)	25.0 (5.5–57.2)	33.3 (9.9–65.1)	18.2 (2.3–51.8)
	*% (95% CI) of participants who complied with the intervention in the workplace*	0.0 (0.0–60.2)	0.0 (0.0–70.8)	0.0 (0.0–70.8)	0.0 (0.0–60.2)	0.0 (0.0–84.2)
e) Satisfaction	*% (95% CI) of participants who perceived the intervention as supportive*	NA	NA	NA	NA	100.0 (71.5–100.0)
f) Return to work	% (95% CI) of participants who had returned/continued to work**	64.3 (35.1–87.2)	69.2 (38.6–90.9)	66.7 (34.9–90.1)	91.7 (61.5–99.8)	90.9 (58.7–99.8)

*NA* not applicable

*There were two dropouts at T0, one at T1, and one at T3. Missing data also occurred, as one participant missed the T2 and T4 appointments but attended the T3 appointment; missing data were not included in the calculation of the percentages of individuals with BC who dropped out from the study

** At T0, T2, T3, and T4, a self-employed worker returned to one of her work activities (two in total). At T1, she was not working at all. At T2, one participant resigned

Adherence to the intervention was above 90% at T1 and T2, decreased at T3 (75.0%), and almost 90% again at T4. Four participants (33.3%) were proposed to organize the intervention at their workplace, but none accepted. As for satisfaction, all participants were satisfied with the intervention received at T4. As for return to work, at T3 and T4, 91.7% (CI 61.5%–99.8%) and 90.9% (CI 58.7%–99.8%) of participants had achieved return to work.

Supplementary material G (p. 8) reports the modules of the UNAMANO project that were offered to support return to work. The majority of the modules were provided at T0, T1, and T3, with occupational therapy being the most frequent. Social support was offered to one participant after she decided to quit her job.

## Discussion

The aim of this study was to verify the feasibility of a multidisciplinary intervention to support the return to work process of individuals with BC by addressing their work difficulties. Feasibility was determined by calculating the percentages of eligibility, recruitment, dropout, adherence to the intervention, satisfaction with the support received, and return to work.

Regarding eligibility and recruitment, a convenience sample of 20–30 individuals with BC was estimated for this prospective feasibility study, based on the initial overestimation aiming to recruit at least 110 patients in the concurrent observational study. Of the cohort of 85 working-age individuals with BC eventually recruited in the observational study, 32 (42.1%) were invited to participate in the feasibility study, and 16 (50% of those eligible) agreed to do so. Although this study did not reach the expected sample size, the recruitment rate compared favorably with that reported in a feasibility study of case management vocational rehabilitation among BC survivors in Scotland (21%) [31], while in The Netherlands, the recruitment percentage of cancer patients in a similar intervention reached 56% [33, 34].

Four participants dropped out from the study, of which two at baseline. Compared with the 20% dropout rate reported in a Dutch feasibility study of a return to work intervention for cancer survivors [35], our study showed a slightly higher dropout rate. This promising result may reflect both the tailored nature of the intervention and the personalized scheduling of appointments. Remote appointments became increasingly frequent from T2 to T4, with minimal change in duration, suggesting sustained engagement despite this modality. Similarly, a feasibility study in Scotland reported telephone appointments as most common modality (79%) [32]. However, the first appointment should be conducted in person, as discussing work-related issues requires time and careful exploration of patients’ perspectives and work environment.

Adherence was high at all assessment appointments, with a minimum of 75% at T3, which is considered favorable for a rehabilitation intervention. A previous online intervention for BC survivors reported adherence rate ranging from 67% to 100% over a 6-week period. However, comparisons between the two studies should be interpreted with caution given the differences in intervention delivery [36].

Information support was mainly provided between T0 and T2, meaning that guidance on work rights seemed most relevant in the early stages return-to-work phase. The most frequent intervention was occupational therapy, encompassing collection of work needs, goal setting, and defining strategies in agreement with participants. In other studies, occupational therapists supported work retention in collaboration with employment counselors [37] or delivered online self-management group interventions for BC survivors [36]. For some participants, the intervention modules ended before study completion, as not all required a 12-month follow-up. The exploratory nature of this intervention has resulted in support exceeding the needs of some participants. Consistent with this interpretation, Leensen et al. (2017) found that some participants did not attend all scheduled counseling sessions with the occupational physician because they had already returned to work or no longer required additional support [34]. Thus, the duration of interventions could be reduced or tailored to participants’ needs in future studies.

The proportion of participants who had returned to work or continued working increased between T0 and T4. Furthermore, all participants assessed at T3 and T4 achieved work reintegration, indicating favorable work-related outcomes over the course of the study. However, current evidence indicates that, although rehabilitation programs may improve perceived work capacity among BC survivors compared with a control group, they do not appear to increase the likelihood of returning to work [38].

Finally, all participants were satisfied with the multidisciplinary intervention. This high level of satisfaction may be attributed to the 12-month duration of the study and the continuous follow-up provided regardless of participants’ employment status, including after they had returned to work, which may have contributed to their positive evaluation of the intervention. Similarly, a feasibility study conducted in Australia evaluating the feasibility of a rehabilitation program to sustain return to work reported high levels of BC survivors’ satisfaction [38]. However, comparison between the two studies should be interpreted with caution as satisfaction was assessed using different measures. In the present study, satisfaction was evaluated using a dichotomous response, whereas the Australian study used a more comprehensive scale, allowing for a more nuanced assessment of participants’ satisfaction across multiple dimensions.

### Strengths and limitations

The strength of the study lies in the tailored nature of the intervention, designed to address each participant’s needs. Consequently, providing a standardized description of specific goals set with each participant and the related strategies is challenging. However, this is in line with the development of complex interventions in health, which require flexibility to address the range of components involved to the behaviors targeted [39]. Moreover, only one participant missed two assessment appointments for organizational reasons unrelated to unwillingness to comply with the intervention.

The recruitment strategy represents the main limitation. Participants were drawn exclusively from a cohort of a concurrent observational study, limiting applicability to routine clinical practice. Moreover, in the observational study, eligibility was restricted to individuals with BC undergoing surgery with lymph node involvement who had attended the educational group session, excluding patients with better or worse prognoses also from the participation in the present study. Consequently, opportunities for participation were not equally distributed, reducing the generalizability of the findings to a broader working-age population. Future studies should adopt more inclusive recruitment strategies, including inpatient settings and the involvement of oncologists and case managers. Additionally, as only about 60% of eligible patients attend the educational session, a substantial percentage of patients could not be offered participation, raising concerns regarding equitable access to supportive interventions [40].

Another limitation is that the estimated sample size was not reached. Nevertheless, adherence, return to work, and satisfaction rates were high. Despite multiple recruitment modalities, participants were selected from an observational study investigating employment outcomes among working-age individuals with BC, suggesting the presence of work-related needs potentially amenable to multidisciplinary support. Participants were likely more willing to reflect on work difficulties and to enroll in this study than those who declined participation in the observational study. Patients who declined, despite being referred to the UNAMANO project, did not contact the occupational therapist and were not recruited in the feasibility study, likely because they had not discussed work-related issues with healthcare professionals, which appeared to be a facilitating factor [23]. Most difficulties and study proposals occurred 7 and 13 months after surgery (follow-up timepoints of the observational study), whereas recruitment peaked at 4 months, possibly reflecting unwillingness to engage in additional research despite the possibility of receiving tailored support.

Finally, results may have been influenced by social desirability bias, whereby participants provide responses perceived as more favorable to the researcher [41]. This bias cannot be excluded since the researcher who followed up the participants was the occupational therapist who delivered the intervention. However, none of the participants reported any aspects that she did not like during the delivery of the intervention.

### Future directions

Future research should investigate the effectiveness of an integrated multidisciplinary approach combining vocational interventions, physical exercise, and psychological support in facilitating the return to work among cancer survivors, in light of the evidence emerging from the most recent literature [19]. Furthermore, the role of occupational therapists should be further investigated as this component was the most frequently delivered within the UNAMANO project. Additionally, appropriate theoretical and practical models should be identified. This study verified the feasibility of a multidisciplinary intervention including occupational therapy, for which a biopsychosocial model like the ICF framework [28] appeared suitable for collecting work-related difficulties.

These findings suggest that the assessment of work-related difficulties and the delivery of supportive interventions may be more appropriate several months after surgery rather than in the immediate postoperative phase, when patients are primarily focused on clinical recovery and prognosis. Notably, nine patients had already returned to work when participation in the feasibility study was proposed. Therefore, future research should confirm this result and explore strategies to support individuals who have not yet resumed work.

As some participants ceased participating in the intervention modules before the study concluded, future research should also define clearer criteria for concluding the intervention, such as achieving predefined goals or completing a specific number of appointments. However, it must be borne in mind that the return-to-work process can change quickly due to the occurrence of unpredictable events in the workplace as well as the fluctuation of patients’ health status.

Finally, further research is needed to determine the effectiveness of this intervention in promoting work participation, not only among individuals with BC but also across different cancer types.

## Conclusions

The aim of this study was to verify the feasibility of the multidisciplinary intervention provided by the social-healthcare pathway of the UNAMANO project. All eligible working-age individuals with BC were recruited from a concurrent observational study. The intervention addressed work-related difficulties experienced by individuals with BC, and both the intervention and the assessment appointments were tailored to participants’ specific needs.

Findings indicate that the intervention was feasible in identifying and recruiting individuals with BC reporting work-related difficulties. Adherence and satisfaction were high, as was the rate of return to work at the end of the intervention, although not all the participants completed the program.

This study provides valuable insights into the feasibility of complex interventions supporting individuals with BC who perceive work-related difficulties. Although complex interventions require flexibility to accommodate the needs of each patient, these findings may inform the design of appropriate interventions, particularly regarding recruitment strategies and timing, intervention characteristics, and the organization and delivery of appointments.

## Supplementary Information

Below is the link to the electronic supplementary material.

ESM 1 Supplementary Material 1 (DOCX 57.2 KB)

## Data Availability

The datasets generated and/or analysed during the current study are available from the corresponding author on reasonable request.
