# Supplementary material for: A multidisciplinary intervention to support return to work of individuals with breast cancer: a prospective feasibility study
Source: Support Care Cancer. 2026 Sep 16;34(10):979. doi: 10.1007/s00520-026-11198-5 (PMC13582325; doi:10.1007/s00520-026-11198-5)
Supplement: Supplementary file 1 [file 520_2026_11198_MOESM1_ESM.docx]

**Supplementary material A.** Sociodemographic, work-related, and disease-related information collected at baseline

The information collected is described below:

a) sociodemographic: sex, age, education level, marital status, children, income range, residence, citizenship.
b) work-related: type of worker, type of company, employment contract, work schedule, night worker, shift worker, flexible work schedule and tasks, psychologically and physically demanding work, number of coworkers, work experience, disability certificate, Law 104 (guarantees legal protection in the workplace according to Italian law in particular concerning health conditions, e.g., paid monthly leave), number of sick days, employment status. Work-related difficulties were categorized through the Core Set-VR-Onco, a checklist based on the International Classification of Functioning, Disability and Health which was recently adapted and validated for a population of cancer survivors of the Province of Reggio Emilia. The ICF has a hierarchical structure comprising components, chapters, and categories. Its four components Body Functions, Body Structures, Activity and Participation, and Environmental Factors are divided into chapters and categories. The Core Set-VR-Onco includes 85 categories: 26 for Body Functions, 33 for Activity and Participation, and 26 for Environmental Factors. Of these, 59 categories represented a problem with respect to the return-to-work process, while the remaining 26 concerned influence on work (dichotomous answer, influenced/did not influence). If the answer was affirmative, it was possible to further distinguish the response as a barrier or a facilitator.
c) disease-related: type of tumor, staging, surgery, removal of sentinel or axillary lymph nodes, chemotherapy, radiotherapy, hormone therapy, targeted therapy, rehabilitation.

**Supplementary material B.** Multidisciplinary intervention

The multidisciplinary intervention was tailored according to individual needs and may include one of the following modules of the UNAMANO project, or a combination of them:

- *information* aims to provide tailored information concerning work legal rights; this support is provided in collaboration with the In-Forma Salute Service of the Azienda USL-IRCCS of Reggio Emilia. The information specialist-nurse responsible for this service provides a wide range of information in response to various requests, including those related to rights at work. At baseline, participants were asked to explain their work difficulties (e.g., not knowing what their work rights were). Information to address these needs was provided immediately after the collection of work difficulties; if the work difficulties were specific to the work sector, the professionals of the social-healthcare pathway took the time to search for the most appropriate information and possibly involved the labor union. After that, the participant was contacted again to provide detailed information. Subsequent assessments were set to monitor the employment status, verify whether participants had complied with what had been agreed on with the social-healthcare pathway, and verify the occurrence of any further work difficulty and/or the need to add other modules.
- *occupational therapy* aims to facilitate the return to work process through the provision of a rehabilitation intervention to overcome work-related difficulties in agreement with the employee, the employer, and the occupational physician. At baseline, participants were asked to describe their work activities, specific tasks, and the amount of time dedicated to each task per day. Subsequently, the tasks in which the patient detected work-related difficulties (e.g., lifting binders, reading a document) were identified and agreed upon, as were the aims of the intervention. The occupational therapy intervention may include work accommodations, tailored strategies for carrying out work tasks, provision of aids or objects to facilitate work task execution, energy conservation techniques, and implementation of a communication plan between employer, employee, and coworkers. Based on a participant’s work-related difficulties and preferences, the occupational therapist may also propose workplace supervision to observe the worker while carrying out work activities. If the worker did not accept workplace supervision, strategies were suggested, but the participant implemented them autonomously in the workplace. If necessary and after obtaining the participant's consent, the occupational therapist may request the support of other professionals of the social-healthcare pathway of the UNAMANO project. For example, a labor union may be activated to provide specific information concerning the participant’s applicable Collective Labor Agreement. Subsequent assessment appointments were set to monitor the employment status and verify whether participants had complied with what had been agreed on with the occupational therapist, verify the occurrence of any further work difficulty, and the need to change the strategies of the intervention provided and/or to add other modules.
- *social support* aims to find new job opportunities for those who have lost their jobs due to cancer. At baseline, data were collected and shared with a social cooperative of the province, which contacted participants to organize an in-person meeting to gather information, assist in job search, prepare a curriculum vitae, conduct skills analysis, and provide professional training. Subsequent assessments monitored employment status and verified compliance with agreements made with the cooperative.

**Supplementary material C.** Recruitment flow diagram

Number of breast cancer survivors in working age recruited in the NewBreca observational study **(n** **= 85)**

Number of patients with work difficulties who were invited to participate in the feasibility study **(n = 32)**

Number of interviews at T4 **(n= 11)**

Number of interviews at T3 **(n= 12)**

Number of interviews at T2 **(n= 12)**

Number of interviews at T1 **(n= 13)**

Number of interviews at T0 **(n= 14)**

Participants who signed the informed consent **(n = 16)**

| **Supplementary material D.** Sociodemographic and disease-related characteristics of 14 participants at T0 | | |
| --- | --- | --- |
|  | | n (%) |
| **Sociodemographic characteristics** | |  |
| *sex* | female | 14 (100.0) |
| *age* | *mean (SD)* | 50.7 (7.0) |
|  | < 50 years | 6 (42.9) |
|  | ≥ 50 years | 8 (57.1) |
| *education level* | middle school | 1 (7.1) |
|  | high school | 10 (71.4) |
|  | university degree | 3 (21.4) |
| *marital status* | married | 6 (42.9) |
|  | divorced | 4 (28.6) |
|  | unmarried | 3 (21.4) |
|  | cohabitant | 1 (7.1) |
|  | widowed | 0 (0.0) |
| *income* | up to €15,000 | 0 (0.0) |
|  | €15,000-€28,000 | 7 (50.0) |
|  | > €28,000-€50,000 | 7 (50.0) |
|  | more than €50,000 | 0 (0.0) |
| *children* | 0 | 3 (21.4) |
|  | 1 | 4 (28.6) |
|  | ≥ 2 | 7 (50.0) |
| *citizenship* | Italian | 12 (85.7) |
|  | Italian and another | 1 (7.1) |
|  | not Italian | 1 (7.1) |
| **Cancer-related characteristics** | |  |
| *type of tumor* | ductal carcinoma | 11 (78.6) |
|  | lobular carcinoma | 2 (14.3) |
|  | other | 1 (7.1) |
| *type of surgery* | conservative | 9 (64.3) |
|  | mastectomy | 5 (35.7) |
|  | axillary lymph node dissection | 4 (28.6) |
| *treatment before surgery* | neoadjuvant chemotherapy | 1 (7.1) |
| **Work-related characteristics** | | |
| *type of worker* | employee | 11 (78.6) |
|  | self-employed | 3 (21.4) |
| *type of company* | private sector | 12 (85.7) |
|  | public sector | 2 (14.3) |
| *type of contract* | permanent | 10 (71.4) |
|  | fixed term | 1 (7.1) |
|  | other | 3 (21.4) |
| *work schedule* | full-time | 9 (64.3) |
|  | part-time | 5 (35.7) |
| *number of workers in the company* | alone | 2 (14.3) |
|  | < 10 | 3 (21.4) |
|  | from 10 to 49 | 1 (7.1) |
|  | from 50 to 249 | 0 (0.0) |
|  | ≥ 250 | 8 (57.1) |
| *work experience (years)* | < 1 | 1 (7.1) |
|  | from 1 to 5 | 3 (21.4) |
|  | from 5 to 10 | 1 (7.1) |
|  | > 10 | 9 (64.3) |
| *shift work* | no | 13 (92.9) |
|  | yes | 1 (7.1) |
| *work on weekends* | no | 12 (85.7) |
|  | yes | 2 (14.3) |
| *work at night* | no | 14 (100.0) |
|  | yes | 0 (0.0) |
| *flexible work schedule* | no | 7 (50.0) |
|  | yes | 7 (50.0) |
| *flexible work tasks* | no | 7 (50.0) |
|  | yes | 7 (50.0) |
| *psychologically demanding job* | no | 4 (28.6) |
|  | sometimes | 1 (7.1) |
|  | yes | 9 (64.3) |
| *physically demanding job* | no | 5 (35.7) |
|  | sometimes | 2 (14.3) |
|  | yes | 7 (50.0) |

Treatments by Follow-Up Timepoint

|  | Chemotherapy | Radiotherapy | Hormone therapy | Targeted therapy | Rehabilitation (physical therapy) |
| --- | --- | --- | --- | --- | --- |
| T4 T0 + 12 months | 0 | 0 | 11 | 0 | 0 |
| T3 T0 + 6 months | 0 | 1 | 11 | 0 | 2 |
| T2 T0 + 3 months | 2 | 1 | 10 | 1 | 2 |
| T1 T0 + month | 3 | 2 | 11 | 2 | 2 |
| T0 baseline | 2 | 8 | 10 | 2 | 3 |

**Supplementary material E.** Absolute number of participants by type of treatment and by each assessment appointment

| **Supplementary material F**. Number of work-related difficulties described through the ICF components and chapters of the CS-VR-Onco by each assessment appointment | | | | | |
| --- | --- | --- | --- | --- | --- |
|  | **T0** | **T1** | **T2** | **T3** | **T4** |
| **BODY FUNCTION** | 14 | 14 | 12 | 14 | 13 |
| b144. Memory functions | 4 | 4 | 4 | 4 | 4 |
| b280. Sensation of pain | 3 | 3 | 2 | 3 | 3 |
| b455. Exercise tolerance functions | 4 | 4 | 4 | 4 | 4 |
| b730. Muscle power functions | 3 | 3 | 2 | 3 | 2 |
| **ACTIVITY AND PARTICIPATION** | 21 | 20 | 18 | 21 | 20 |
| d160. Focusing attention | 2 | 2 | 2 | 2 | 2 |
| d210. Undertaking a single task | 2 | 2 | 2 | 2 | 2 |
| d220. Undertaking multiple tasks | 3 | 2 | 2 | 2 | 2 |
| d240. Handling stress and other psychological demands | 2 | 1 | 1 | 1 | 1 |
| d415. Maintaining a body position | 1 | 1 | 1 | 1 | 1 |
| d430. Lifting and carrying objects | 3 | 3 | 2 | 3 | 3 |
| d445. Hand and arm use | 2 | 2 | 1 | 2 | 1 |
| d475. Driving | 0 | 0 | 0 | 0 | 1 |
| d850. Remunerative employment | 4 | 5 | 5 | 6 | 5 |
| d870. Economic self-sufficiency | 2 | 2 | 2 | 2 | 2 |
| **ENVIRONMENTAL FACTORS** | 13 | 13 | 13 | 13 | 11 |
| e115. Products and technology for personal use in daily living | 1 | 0 | 0 | 0 | 0 |
| *Facilitators* | 1 | 0 | 0 | 0 | 0 |
| *Barriers* | 0 | 0 | 0 | 0 | 0 |
| e135. Products and technology for employment | 2 | 2 | 2 | 2 | 2 |
| *Facilitators* | 2 | 2 | 2 | 2 | 2 |
| *Barriers* | 0 | 0 | 0 | 0 | 0 |
| e325. Acquaintances, peers, colleagues, neighbours and community members | 2 | 3 | 3 | 3 | 3 |
| *Facilitators* | 2 | 3 | 3 | 3 | 3 |
| *Barriers* | 0 | 0 | 0 | 0 | 0 |
| e330. People in positions of authority | 3 | 3 | 3 | 4 | 3 |
| *Facilitators* | 2 | 2 | 2 | 3 | 3 |
| *Barriers* | 1 | 1 | 1 | 1 | 0 |
| e425. *Individual attitudes of acquaintances, peers, colleagues, neighbors and community members* | 1 | 1 | 1 | 1 | 1 |
| *Facilitators* | 1 | 1 | 1 | 1 | 1 |
| *Barriers* | 0 | 0 | 0 | 0 | 0 |
| e570. Social security services, systems and policies | 1 | 1 | 1 | 1 | 1 |
| *Facilitators* | 0 | 0 | 0 | 0 | 0 |
| *Barriers* | 1 | 1 | 1 | 1 | 1 |
| e580. Health services, systems and policies | 1 | 1 | 1 | 1 | 0 |
| *Facilitators* | 0 | 0 | 0 | 0 | 0 |
| *Barriers* | 1 | 1 | 1 | 1 | 0 |
| e590. Labour and employment services, systems and policies | 2 | 2 | 2 | 2 | 1 |
| *Facilitators* | 0 | 0 | 0 | 0 | 0 |
| *Barriers* | 2 | 2 | 2 | 2 | 1 |

| **Supplementary G.** Modules of the UNAMANO project that were offered to support return to work by each assessment appointment | | | | | | |
| --- | --- | --- | --- | --- | --- | --- |
|  | **T0** | **T1** | **T2** | **T3** | **T4** | **total** |
| **Information** | 4 | 3 | 1 | 0 | 0 | 8 |
| **Occupational Therapy** | 8 | 7 | 6 | 7 | 6 | 34 |
| **Labor Union** | 2 | 2 | 3 | 1 | 1 | 9 |
| **Social Support** | 0 | 0 | 1 | 0 | 0 | 1 |
| **Support ended** | 1 | 1 | 2 | 4 | 4 | 12 |
| **Total** | 15 | 13 | 13 | 12 | 11 |  |
